# The Prediction Potential of the Pretreatment Lung Immune Prognostic Index for the Therapeutic Outcomes of Immune Checkpoint Inhibitors in Patients With Solid Cancer: A Systematic Review and Meta-Analysis

**DOI:** 10.3389/fonc.2021.691002

**Published:** 2021-09-23

**Authors:** Hui Liu, Xiao-Li Yang, Xiao-Yun Yang, Zhao-Ru Dong, Zhi-Qiang Chen, Jian-Guo Hong, Tao Li

**Affiliations:** ^1^ Department of General Surgery, Qilu Hospital, Shandong University, Jinan, China; ^2^ Department of Nephrology, Jinan Central Hospital, Shandong University, Jinan, China; ^3^ Department of Gastroenterology, Qilu Hospital, Shandong University, Jinan, China; ^4^ Department of Hepatobiliary Surgery, The Second Hospital of Shandong University, Jinan, China

**Keywords:** lung immune prognostic index, immune checkpoint inhibitors, solid cancer, chemotherapy, meta-analysis

## Abstract

**Background:**

The lung immune prognostic index (LIPI) is recently developed to predict immune checkpoint inhibitors (ICIs) treatment outcomes for non-small cell lung cancer. However, its predictive value for other types of cancer remained unclear. This meta-analysis aimed to evaluate the association between pretreatment LIPI score and therapeutic outcomes in cancer patients treated with ICIs.

**Methods:**

We searched PubMed, Cochrane Library literature databases and EMBASE for abstracts and full-text articles published from the inception of the database until 16th, Nov 2020. Meta-analyses were performed separately for progression-free survival (PFS) and overall survival (OS) by using the random-effects model.

**Results:**

A total of 12 studies involving 4883 patients receiving ICIs treatment were identified for the primary analysis. The pooled results implied that compared with good LIPI score groups, patients with poor or intermediate LIPI score were significantly associated with worse OS (HR=3.33, 95%CI 2.64-4.21, P < 0.001, I^2^ = 64.2%; HR=1.71, 95%CI 1.43-2.04, P < 0.001, I^2^ = 43.6%, respectively) and PFS (HR=2.73,95%CI 2.00-3.73, P < 0.001, I^2^ = 78.2%; HR=1.43, 95%CI 1.28-1.61, P < 0.001, I^2^ = 16.3%, respectively). Also, for 1873 patients receiving chemotherapy, a poor LIPI score was significantly associated with worse OS (HR=2.30, 95%CI 1.73-3.07, P < 0.001; I^2^ = 56.2%) and PFS (HR=1.92,95%CI 1.69-2.17; P < 0.001; I^2^ = 0.0%) compared with good LIPI score groups.

**Conclusions:**

A good LIPI score was significantly correlated with improved OS and PFS in cancer patients receiving ICIs or chemotherapy, regardless of the types of cancer.

## Introduction

Immune checkpoint inhibitors (ICIs) mainly include antibodies against cytotoxic T lymphocyte-associated protein 4 (CTLA-4), programmed cell death 1 (PD-1), and its major ligand (PD-L1) (1). ICIs have marked efficacy in the treatment of patients with solid cancers (2–4), still the majority of patients show intrinsic resistance to ICIs treatment owing to the tumor microenvironment (TME) and an impaired T cell tumor interaction mediated by immune escape mechanisms of tumor and immune cells (5). Therefore, resistance to ICIs treatment restricts patients with advanced cancer to achieve durable responses.

There are no candidate biomarkers for predicting response or resistance to immunotherapy in solid cancers, including hepatocellular carcinoma (6). Biomarkers that can predict whether patients have long-term favorable respond to ICIs therapy are eagerly awaited. At present, several biomarkers have been recognized to be associated with clinical outcomes for ICIs treatment. PD-L1, PD-1, neutrophil-lymphocyte ratio (NLR), tumor-infiltrating lymphocyte (TIL), microsatellite instability (MSI), gene expression profiling (GEP), and tumor mutational burden (TMB) can improve the predictive accuracy for ICIs outcomes in solid cancers (7–10).

The lung immune prognostic index (LIPI) was developed on the basis of derived neutrophils/(leukocytes minus neutrophils) ratio (dNLR) greater than three and lactate dehydrogenase (LDH) greater than upper limit of normal (ULN), characterizing three groups (good LIPI score group, 0 factor; intermediate LIPI score group,1 factor; poor LIPI score group, 2 factors) (11). Current evidence proved that the LIPI score could be used to identify cancer patients who benefit from ICIs treatment in multiple cancers (11, 12). Meanwhile, contradictory results have also been published (13, 14).

Herein, we performed a systematic review and meta-analysis, to investigate the significance of the LIPI score as a predictive tool in solid cancer patients receiving ICIs treatment based on 12 published studies.

## Methods

This meta-analysis was performed by the Preferred Reporting Items for Systematic reviews and Meta-Analyses (PRISMA) guidelines (15), with inclusion criteria being set out according to the PICOS model.

### Search Methods and Study Selection Criteria

Two authors (HL, XLY) independently searched relevant studies from the PubMed, EMBASE, and Cochrane Library literature databases from the inception of the database until 16th, Nov 2020. Those studies were restricted to the English language. The following search retrieval keywords for the literature were employed, including neoplasms, cancer, lung immune prognostic index, LIPI, PD-1, PD-L1, CTLA-4, nivolumab, atezolizumab, avelumab, durvalumab, ipilimumab, tremelimumab, pembrolizumab, ICIs, immune checkpoint inhibitor. The detailed search strategy is presented in **Supplementary Retrieval Methods**.

Two investigators (HL and XYY) independently screened the literature, and discrepancies were reviewed by another investigator on the team (TL) and resolved by consensus. The main criteria used for the eligibility study were as follows: (1) the studies in which patients were histologically diagnosed with cancer and treated with ICIs or chemotherapy. (2) the studies where the association between LIPI and therapeutic outcomes such as progression-free survival (PFS), overall survival (OS) were evaluated. (3) the studies where the related data could be collected directly or calculated indirectly. (4) the studies provided sufficient information to assess hazard ratio (HR) with 95% confidence interval (95%CI). (5) the studies that were published in English. For re-published research, only the latest literature and relevant data can be collected, or the research with the largest sample size can be selected.

### Data Extraction and Quality Assessment

Two researchers (HL and XLY) independently performed the following data from each study: (1) first author, publication year, country, sample size, immune checkpoint inhibitors, study design, follow-up time. (2) the outcome measures (OS, PFS) and HR with 95% CI extracted from original studies. When both univariate analysis and multivariate analysis are available, we give priority to the results in multivariate analysis. If the information we needed was unreported or unable to be calculated indirectly, or not available after contacting the corresponding authors, the study will be used for systematic review or discarded.

The quality of studies will be evaluated by the Newcastle-Ottawa Scale (NOS) criteria (16). Studies with a score greater than seven were considered as high-quality literature, studies with a score of five to seven were considered as medium-quality literature, studies with a score less than five were considered as poor-quality literature.

### Statistical Analysis

Data analyses were performed through Stata 12.0 statistical software (Stata Corp LP, College Station, TX), P < 0.05 was considered statistically significant. OS and PFS were used to evaluate the correlation between LIPI score and clinical data of cancer patients receiving ICIs or chemotherapy. HR with 95%CI was used for the pooled analyses of OS and PFS.

We compared the good LIPI score groups with the poor LIPI score, and intermediate LIPI score groups. Intermediate/poor (intermediate + poor) LIPI score groups were also compared with good LIPI score groups due to the combination of intermediate and poor LIPI score groups. Subgroup analyses were performed by cancer type, sample size, study region. At the same time, we also evaluated the association between different LIPI score and cancer patients receiving chemotherapy. Meta-analysis was used to pool the estimates, using the random-effects model.

Statistical heterogeneity was evaluated by using the Cochran Q and the inconsistency index (I^2^) statistic tests. P > 0.05 and I^2^ < 50% indicated a lack of heterogeneity among all studies. We chose the random effect model in this meta-analysis due to the inherent clinical heterogeneity among studies included in this meta-analysis (17). Funnel plots, Egger’s regression asymmetry test, and Begg’s rank correlation test were used to examine the potential publication bias (18).

## Results

### Retrieval Result and Study Characteristics

**Figure 1:**
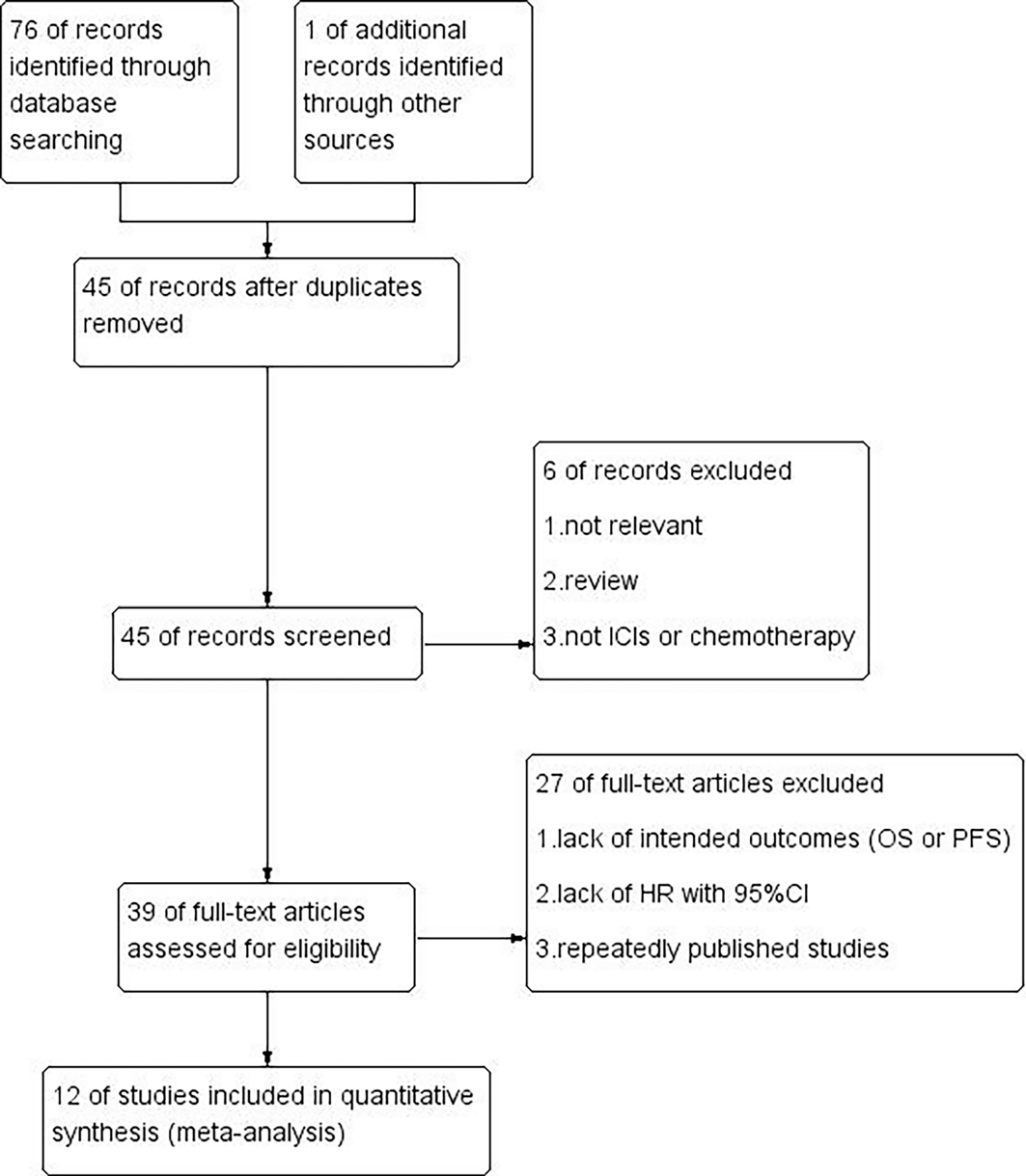
Flow diagram of study selection.

A flow chart of the literature search process is summarized in **Figure 1**. In total, 76 records were initially identified. Then, 45 studies were retained after the removal of duplicates. After screening for titles and abstracts, 6 records were excluded (not relevant, review, not ICIs or chemotherapy). After reviewing the remaining 39 articles *via* the full-text view, 27 full-text articles were excluded due to lack of intended outcomes (OS, PFS), HR with 95% CI or repeatedly published studies. Finally, 12 studies published between 2018 and 2020 were included in this analysis (11–14, 19–26).

The detailed characteristics of all eligible studies are presented in **Table 1**. One of twelve studies was prospectively designed; the rest of the literature was retrospectively designed. In total, our meta-analysis included 4883 cancer patients receiving ICIs treatment. The sample size was ranging from 70 to 1489 per study. Eight studies were conducted in Europe; two were conducted in China; one was from Australia; one was conducted in Canada; a conference abstract was not reported. Among these studies, seven studies included patients with non-small cell lung cancer (NSCLC), one for hepatocellular carcinoma and one with squamous cell carcinoma of the head and neck (SCCHN); the rest four studies for other types or multiple cancers. All patients enrolled were treated with ICIs or chemotherapy. The quality assessment results of the all 12 studies ranged from 5 to 7, as shown in **Table 1**. Four studies scored 7 points, seven studies scored 6 points, and one study scored 5 points.

**Table 1:** The characteristics of included studies.

Author	Study period	Data collection	Country	Cancer type	ICIs	Sample size	Outcome	Median follow-up (months)	NOS
Wang	2016-2019	retrospective	China	NSCLC	NR	216	PFS,OS	NR	6
Sorich	NR	retrospective	Australia	NSCLC	Atezo	1489	PFS,OS,ORR	15.1 (14.7-15.4)	7
Mielgo	2015-2019	retrospective	Spain	NSCLC	Pembro	223	PFS,OS,DCR	NR	6
Meyers	2010-2019	retrospective	Canada	Multiple	Nivo, Pembro, Ipi/Nivo	578	PFS,OS,ORR	23.5 (1.8-89.0)	7
Mazzaschi	2015-2019	prospective	Italy	NSCLC	Nivo,Pembro,Atezo	109	PFS,OS	17.3	7
Kazandjian	2013-2017	retrospective	Europe	mNSCLC	Atezo, Nivo, Pembro	1368	PFS,OS	NR	6
Herrera	2014-2019	retrospective	Europe	SCCHN	ICIs	190	PFS,OS,ORR	13.2	6
Ferreira	2015-2019	retrospective	Portugal	lung cancer	Pembro,Nivo	120	OS	13	6
Chen	2015-2019	retrospective	China	aHCC	Nivo,Pembro	108	PFS,OS	NR	6
AI Darazi	2015-2018	retrospective	France	Multiple	ICIs	259	OS	15 (11.6-17.5)	7
Santa	2015-2019	prospective	NR	Multiple	ICIs	70	PFS,OS,ORR	NR	6
Ruiz-Bañobre	2015-2017	retrospective	Spain	NSCLC	Nivo	153	PFS,OS,DCR	NR	5

NSCLC, Non-small Cell Lung Cancer; Atezo, atezolizumab; Nivo, nivolumab; Pembro, pembrolizuma; Ipi, Ipilimumab; ORR, objective response rate; DCR, disease control rate; NR, not reported; ICIs, immune checkpoint inhibitors; SCCHN, head and neck squamous cell carcinoma.

### Association Between LIPI Score and OS or PFS of Cancer Patients Receiving ICIs Treatment

**Figure 2:**
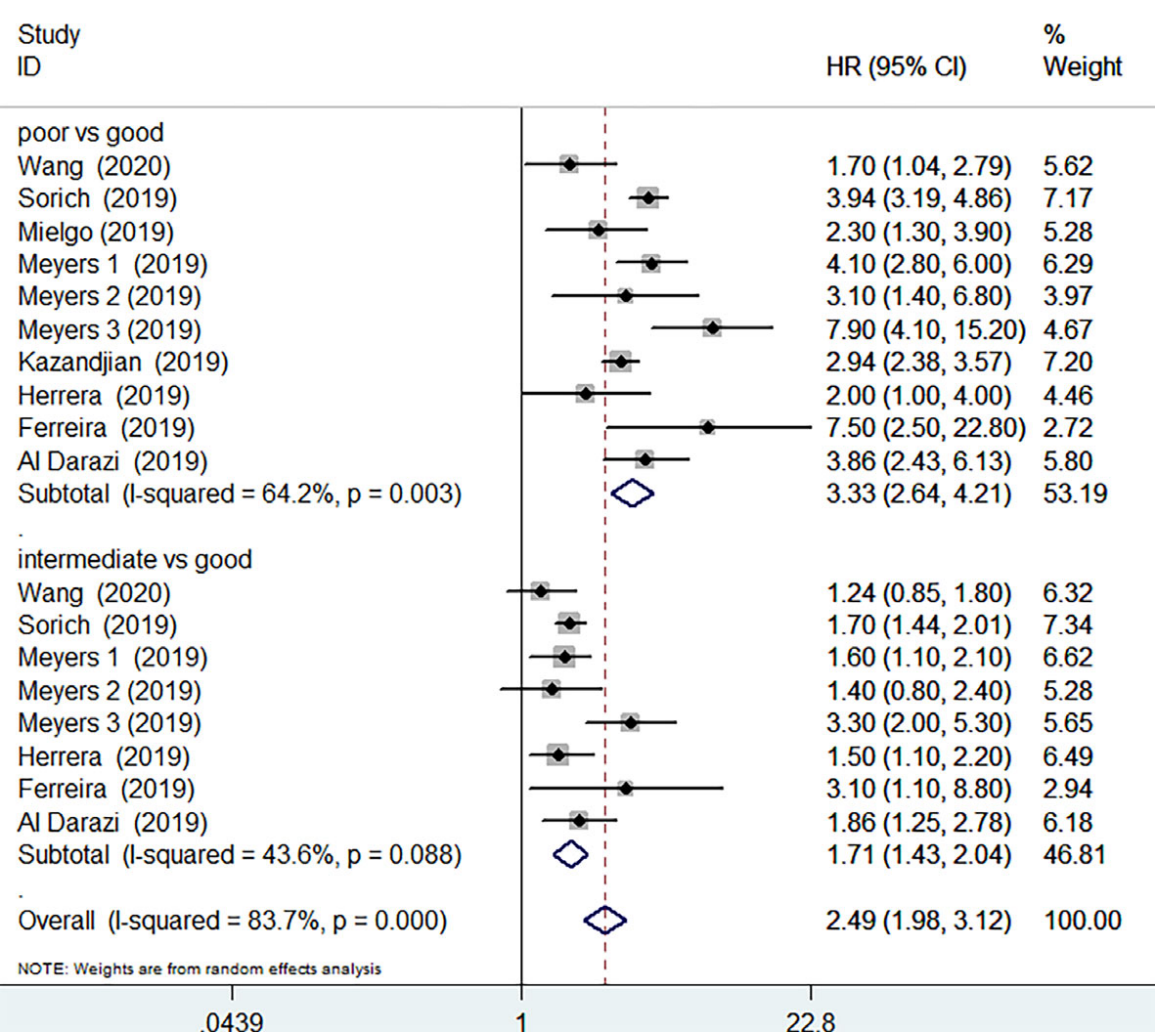
Meta-analysis of OS in cancer patients after ICIs treatment (poor LIPI score groups *vs.* good LIPI score groups, intermediate LIPI score groups *vs.* good LIPI score groups).

OS data was available in 8 studies involving 4443 patients receiving ICIs treatment. Compared with good LIPI score groups, poor LIPI score groups were significantly associated with worse OS (HR=3.33, 95%CI =2.64-4.21; P< 0.001), with a significant level of heterogeneity (I^2^ = 64.2%, P=0.003) among the studies (**Figure 2**). Intermediate LIPI score predicted poor OS compared with good LIPI score groups (HR=1.717, 95%CI 1.43-2.04; P< 0.001) (**Figure 2**) with a moderate level of heterogeneity (I^2^ = 43.6%, P=0.088). The sensitivity analysis showed that the pooled OS results (**Supplementary Figures 1** and **2**) were not significantly changed by any single study.

**Figure 3:**
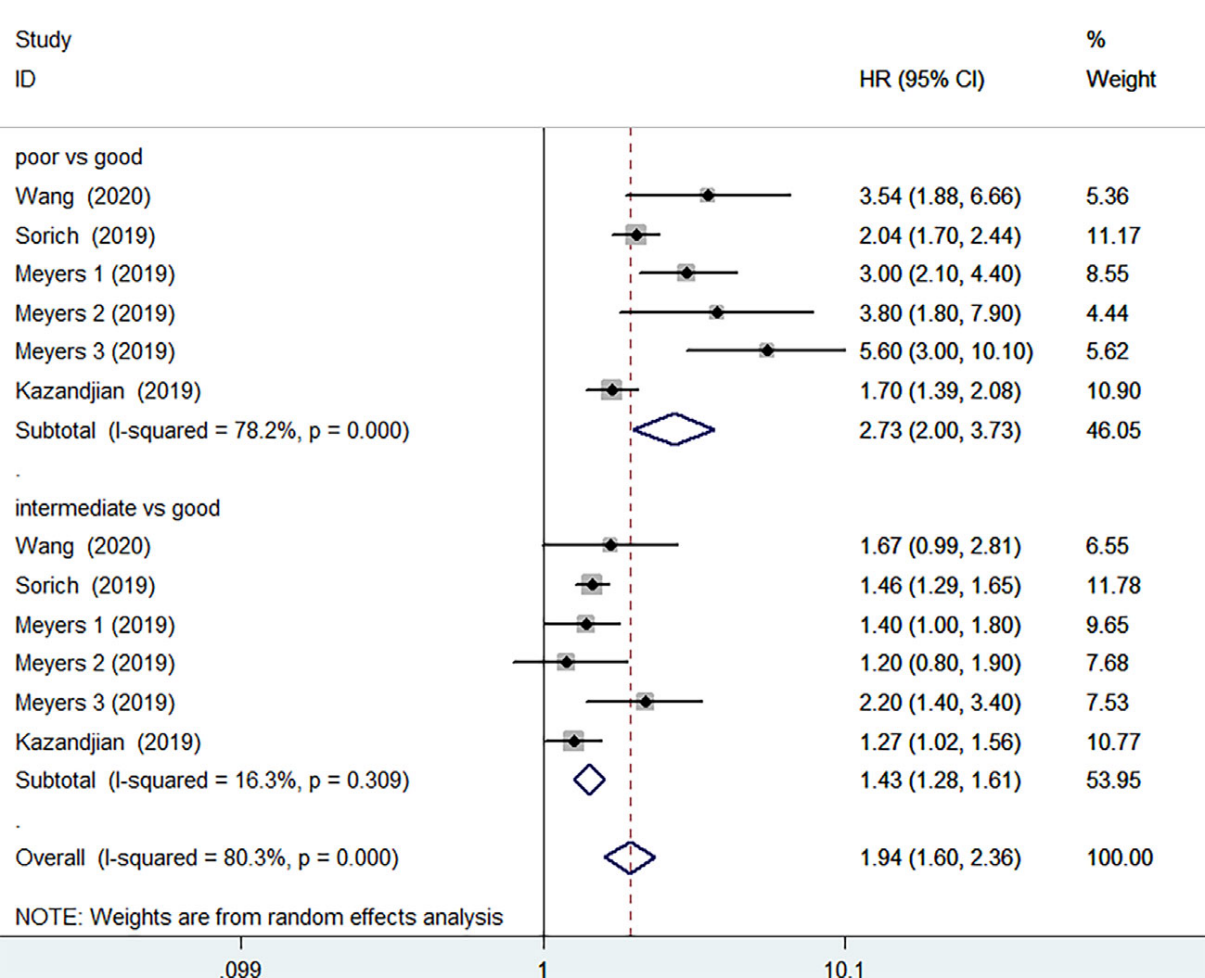
Meta-analysis of PFS in cancer patients after ICIs treatment (poor LIPI score groups *vs.* good LIPI score groups, intermediate LIPI score groups *vs.* good LIPI score groups).

PFS data was available in 4 studies involving 3669 patients. The pooled results implied that poor LIPI score groups had a significantly higher risk of poor PFS compared with good LIPI score groups (HR =2.73, 95%CI 2.00-3.73, P < 0.001). The heterogeneity test showed significant heterogeneity existed among these studies (I^2^ = 78.2%, P< 0.001) (**Figure 3**). The pooled results revealed that intermediate LIPI score indicated poor PFS compared with good LIPI score groups (HR=1.43, 95%CI 1.28-1.61, P< 0.001) with a moderate level of heterogeneity (I^2^ = 16.3%, P=0.309) (**Figure 3**).

**Figure 4:**
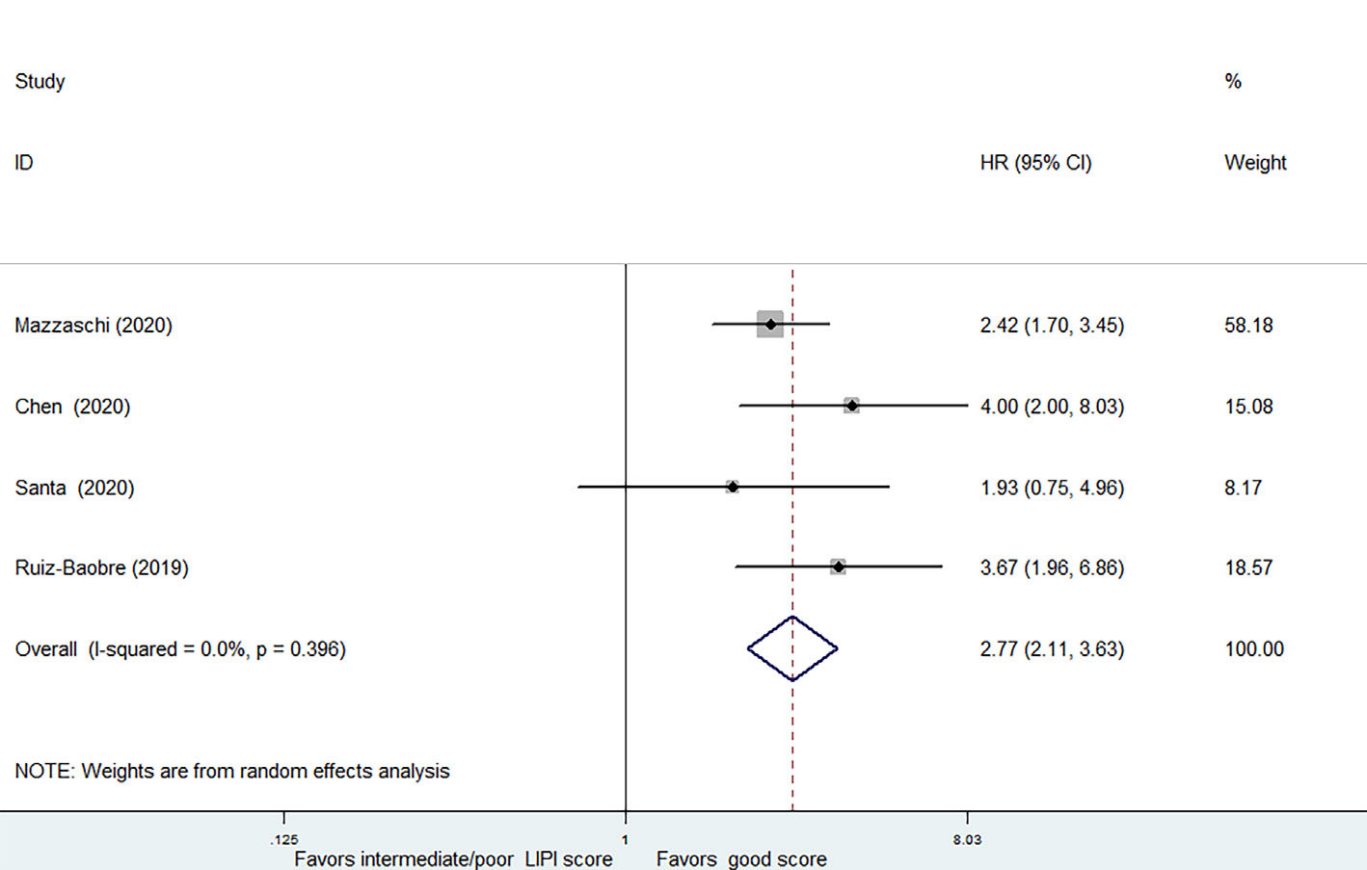
Meta-analysis of OS in cancer patients after ICIs treatment (intermediate + poor LIPI score groups *vs.* good LIPI score groups).

4 studies, covering 440 patients receiving ICIs treatment, were analyzed separately due to the combination of intermediate and poor LIPI score groups (intermediate/poor LIPI score groups). Significantly worse OS was also found in intermediate/poor LIPI score groups than good LIPI score groups (HR=2.77, 95%CI 2.11-3.63, P < 0.001), without any heterogeneity (I^2^ = 0.0%, P=0.396) (**Figure 4**). PFS data was available in 3 studies involving 370 patients receiving ICIs. Intermediate/poor LIPI score groups had inferior PFS (HR=2.13, 95%CI 1.55-2.93; P<0.001) compared with good LIPI score group. The heterogeneity test showed moderate heterogeneity existed among these studies (I^2^ = 41.7%, P=0.18) (**Supplementary Figure 3**). The sensitivity analysis also showed that no individual research influenced the pooled effects on PFS (**Supplementary Figures 4** and **5**).

### Association Between LIPI Score and OS or PFS of Cancer Patients Receiving Chemotherapy

**Figure 5:**
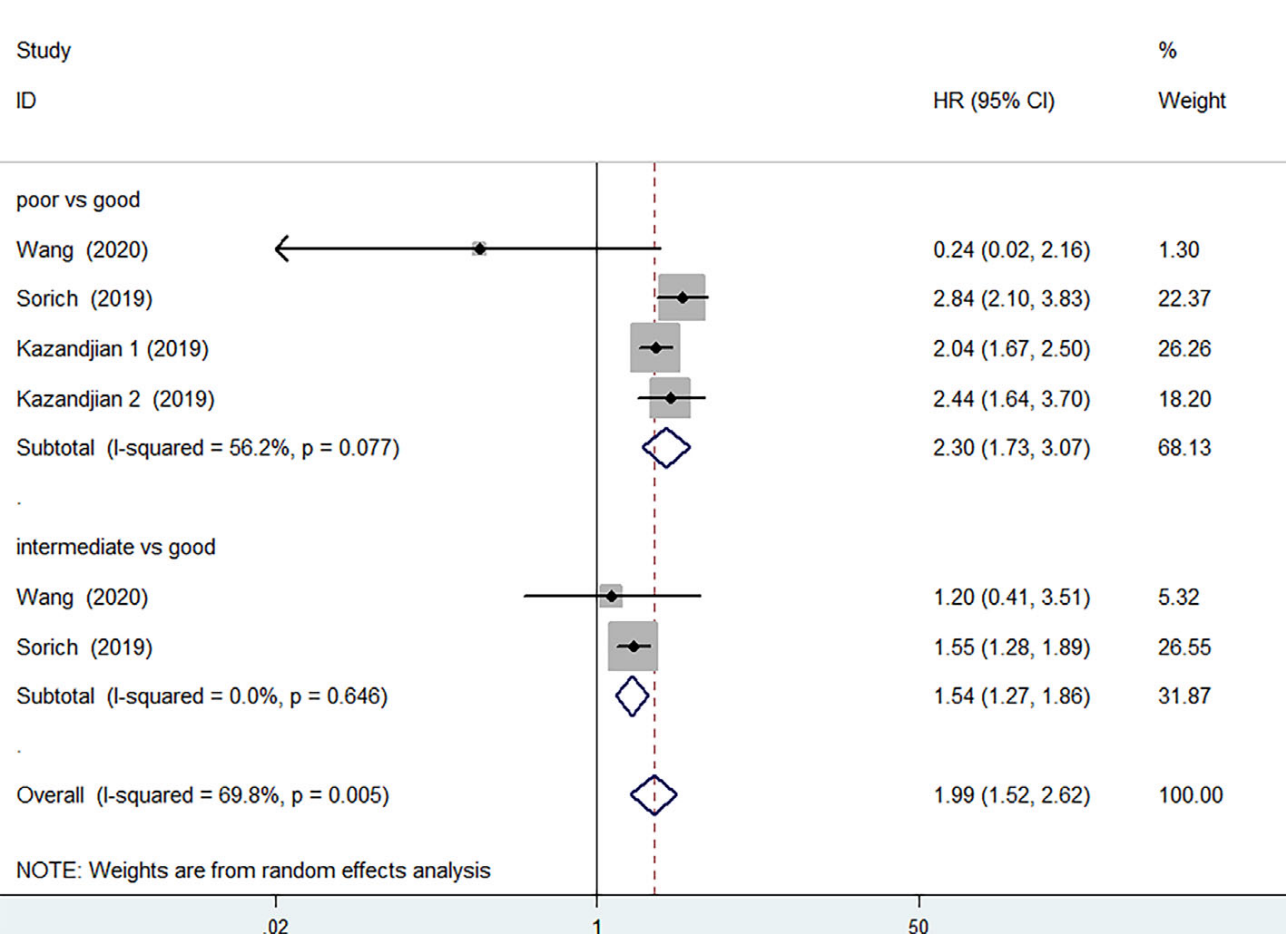
Meta-analysis of OS in cancer patients after chemotherapy (poor LIPI score groups *vs.* good LIPI score groups, intermediate LIPI score groups *vs.* good LIPI score groups).

**Figure 6:**
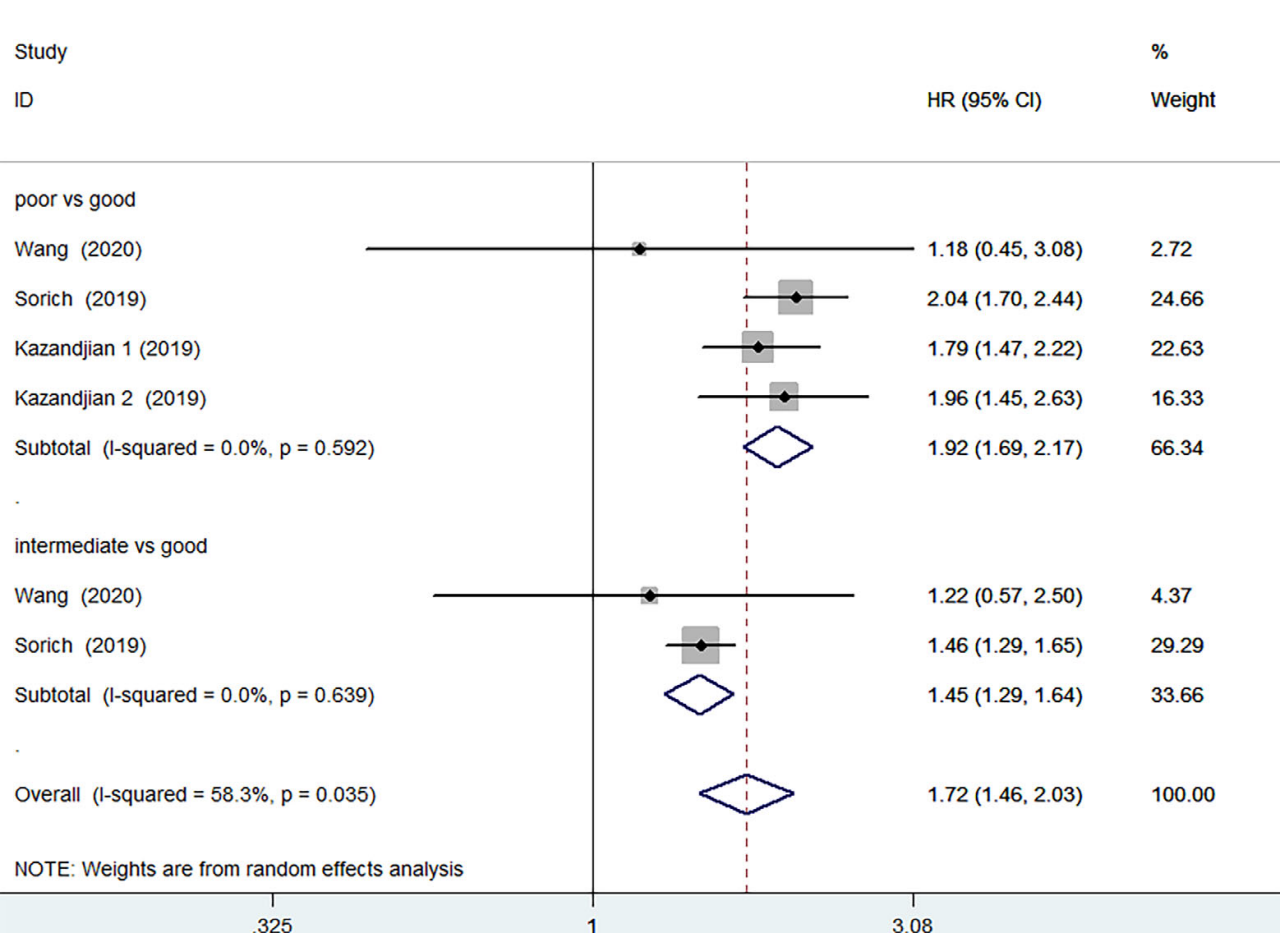
Meta-analysis of PFS in cancer patients after chemotherapy (poor LIPI score groups *vs.* good LIPI score groups, intermediate LIPI score groups *vs.* good LIPI score groups).

3 of the 12 studies involving 1873 patients receiving chemotherapy provided OS and PFS data. According to the random-effects model, compared with the group with good LIPI score, the group with poor LIPI score was significantly correlated with the group with inferior OS (HR=2.30, 95%CI 1.73-3.07, P < 0.001) (**Figure 5**) and PFS(HR=1.92, 95%CI 1.69-2.17; P < 0.001) (**Figure 6**). The heterogeneity test showed that heterogeneity existed among these studies in OS (I^2^ = 56.2%, P=0.077), but not PFS (I^2^ = 0.0%, P=0.592). Intermediate LIPI score was also a significant association with worse OS (HR=1.54, 95%CI 1.27-1.86, P < 0.001) (**Figure 5**) and PFS (HR=1.45, 95%CI 1.29-1.64; P < 0.001) (**Figure 6**) compared with good LIPI score groups. Neither of them is heterogeneous (I^2^ = 0.0%, P=0.646; I^2^ = 0.0%, P=0.639). However, the LIPI score may not predict therapeutic outcomes in cancer patients receiving chemo-immunotherapy (26). The sensitivity analysis also showed that no individual study influenced the pooled effects on OS and PFS (**Supplementary Figures 6** and **7**).

### Association Between Subgroup Analyses and OS or PFS

Subgroup analyses for OS and PFS were further performed to assess the interaction of LIPI score with cancer type, sample size, and study region and the results are shown in **Table 2**. When stratifying by cancer type, compared with the good LIPI score groups, poor LIPI score was significantly associated with worse OS and PFS in patients with NSCLC (OS: HR=3.011, 95%CI 2.303-3.936; P<0.001) (PFS: HR=3.011, 95%CI 2.303-3.936; P<0.001) and multiple/other (OS: HR=4.107, 95%CI 2.525-60681; P<0.001) (PFS: HR=4.791, 95%CI 2.997-7.659; P<0.001) (**Table 2**). The conclusion was the same when stratifying by sample size and study region. There was significant heterogeneity in the sample size less than 200 (I^2^ = 82.8%, P=0.001), NSCLC (I^2^ = 70.8%, P =0.003) and other study region (I^2^ = 89.4%, P=0.002).

**Table 2:** Subgroup analyses of the associations between LIPI score and outcomes (poor *vs.* good).

	Subgroup	Number of studies	Pooled results		Heterogeneity test		Publication Bias test		
			HR (95CI %)	P	I2	P	P (Begg’s)		P (Egger’s)
**Overall survival**	All studies	10	3.332 (2.639-4.208)	<0.001	64.2	0.003	0.859	0.939	
Cancer type	NSCLC	5	3.011 (2.303-3.936)	<0.001	70.8	0.008	0.462	0.376	
	Multiple/other	5	4.107 (2.525-60681)	<0.001	58.5	0.047	1	0.75	
Sample size	<200	4	3.571 (1.547-8.244)	0.003	82.8	0.001	0.734	0.388	
	>200	6	3.416 (2.892-4.036)	<0.001	29.2	0.216	0.858	0.939	
Study region	Canada	3	4.659 (2.896-7.496)	<0.001	48.9	0.141	1	0.874	
	Europe	5	3.001 (2.301-3.914)	<0.001	33.6	0.198	1	0.747	
	Others	2	2.669 (1.174-6.069)	0.019	89.4	0.002	1	0.219	
**Progression-free survival**	All studies	6	2.733 (2.000-3.733)	<0.001	78.2	<0.001	0.707	0.021	
Cancer type	NSCLC	4	2.242 (1.717-2.929)	<0.001	70.9	0.016	0.734	0.182	
	Multiple/other	2	4.791 (2.997-7.659)	<0.001	0.0	0.427	1		
Sample size	<200	2	4.492 (2.867-7.039)	<0.001	4.9	0.305	1		
	>200	4	2.232 (1.704-2.922)	<0.001	70.3	0.018	0.737	0.198	
Study region	Canada	3	3.760 (2.562-5.520)	<0.001	33.1	0.224	1	0.481	
	Others	2	2.464 (1.476-4.114)	<0.001	62.9	0.1			

LIPI, lung immune prognostic index; NSCLC, non-small cell lung cancer; HR, hazard ratio.

### Publication Bias

The funnel plot indicated no significant publication bias in all the pooled analyses (**Supplementary Figures 8** and **9**). Funnel plot asymmetry was further assessed by the method of Egger’s and Begg’s linear regression test (P < 0.05 was considered a significant publication bias) (18). The Begg’s and Egger’s test also revealed no evidence of publication bias for OS (Begg’s test: P = 0.534, Egger’s test: P = 0.536) and PFS (Begg’s test: P = 0.707, Egger’s test P = 0.021) (**Supplementary Figures 10** and **11**). We did not conduct a publication bias analysis for those with too few studies.

## Discussion

The LIPI score is developed based on LDH and dNLR, which are simple and easy to calculate (11). In this meta-analysis, we have evaluated the association of LIPI score with the prognosis of cancer patients receiving ICIs or chemotherapy. Compared with good LIPI score groups, the pooled results show that regardless of whether the patients received ICIs or chemotherapy, the OS and PFS of the poor or intermediate LIPI score group were significantly reduced. Although some studies have shown that the LIPI score cannot be used to identify cancer patients who have benefited from chemotherapy, these data are not available (11).

Inflammation plays a key role in tumor progression, affecting the survival of cancer patients (27). The inflammatory status of various cancers can be measured by dNLR. As an essential component of the inflammatory response, neutrophils not only target tumor cells, but also indirectly act on the tumor microenvironment to promote tumor development (28). Lymphocyte infiltration in the tumor is also associated with a better response to immunotherapy and prognosis in solid tumor patients (29). LDH and dNLR are independent risk factors for mortality among patients, and are correlated with poor outcomes in several solid cancer types according to the current evidence (30, 31).

Immunotherapy has demonstrated great clinical success in certain cancers, but so far, immunotherapy has only achieved success in a limited number of cancers (32). Specifically, Callahan, M.K., et al. have demonstrated that the majority of such patients can now be successfully treated with concurrent ipilimumab and nivolumab therapy (33). However, the deleterious effects of immune-related adverse events (irAEs) might outweigh the benefit from the addition of ipilimumab (34). Until now, there is still no biomarker for some cancers to predict the response or resistance to immunotherapy (6). Robust biomarkers are needed to predict patient responsiveness to immunotherapy and for their stratification (35). Our meta-analysis provides new evidence supporting that LIPI scores are used as prognostic indicators for cancer patients receiving ICIs or chemotherapy. In addition, LIPI score may also has prognostic value for doublet immunotherapy such as ipilimumab and nivolumab, as one included study has revealed the potential application of the LIPI scores in such circumstances (12). However, more studies are warranted to fully exploit its predictive value.

Our findings suggest that the LIPI scores may be a promising predictive biomarker for the therapeutic outcomes of ICIs or chemotherapy in solid cancer patients. The pooled results for subgroup analyses, which involved types of cancer, sample size, and study regions, indicated that there is significant heterogeneity in the sample size less than 200, and other study regions (including Australia and China). Previously, the LIPI score may be an independent and complemented indicator for improved survival in cancer patients receiving immunotherapy or chemotherapy, because there was no significant correlation among PD-L1, TMB, and LIPI score (36).

Although our analysis provided a comprehensive summary of current literature, our present study still had some limitations that need to be considered. First of all, the major limitation lies in the fact that most of the included studies were retrospective, leading to some unavoidable bias sources. Secondly, only studies published in English were included, which may lead to publication bias. Cancer type is also a limitation; thus, we performed a subgroup analysis to further explain the correlation between LIPI score and the clinicopathologic features of patients receiving ICIs or chemotherapy. Although there is significant heterogeneity when the sample size is less than 200, this does not affect our results. Further high-quality prospective study is warranted.

## Conclusions

In summary, a good LIPI score was significantly correlated with improved OS and PFS in cancer patients receiving ICIs or chemotherapy, regardless of the different types of cancer. Our analysis supported that the LIPI score is a reliable predictive tool that can be used to identify cancer patients who benefit from ICIs or chemotherapy, and to guide decisions in the era of personalized cancer treatments.

## Data Availability Statement

The original contributions presented in the study are included in the article/**Supplementary Material**. Further inquiries can be directed to the corresponding author.

## Author Contributions

HL and TL designed the study. HL, X-LY, and Z-RD performed the systematic search. HL, X-LY, X-YY, Z-RD, and Z-QC selected eligible articles and conducted the quality assessment. HL and X-LY analyzed, interpreted the data, and drafted the manuscript. TL revised the manuscript. All authors contributed to the article and approved the submitted version.

## Funding

This work was supported by the grants from the Taishan Scholars Program for Young Expert of Shandong Province (Grant No. tsqn20161064), National Natural Science Foundation of China (Grant No. 82073200 & 81874178), and founds for Independent Cultivation of Innovative Team from Universities in Jinan (Grant No. 2020GXRC023).

## Conflict of Interest

The authors declare that the research was conducted in the absence of any commercial or financial relationships that could be construed as a potential conflict of interest.

## Publisher’s Note

All claims expressed in this article are solely those of the authors and do not necessarily represent those of their affiliated organizations, or those of the publisher, the editors and the reviewers. Any product that may be evaluated in this article, or claim that may be made by its manufacturer, is not guaranteed or endorsed by the publisher.

## Abbreviations

LIPI, lung immune prognostic index; ICIs, immune checkpoint inhibitors; PFS, progression-free survival; OS, overall survival; CTLA-4, cytotoxic T lymphocyte-associated protein 4; PD-1, programmed cell death 1; TME, tumor microenvironment; NLR, neutrophil-lymphocyte ratio; TIL, tumor-infiltrating lymphocyte; MSI, microsatellite instability; GEP, gene expression profiling; TMB, tumor mutational burden; LDH, lactate dehydrogenase; ULN, upper limit of normal; NSCLC, non-small cell lung cancer; SCCHN, squamous cell carcinoma of the head and neck; irAEs, immune-related adverse events.
