# Supplementary material for: The Prediction Potential of the Pretreatment Lung Immune Prognostic Index for the Therapeutic Outcomes of Immune Checkpoint Inhibitors in Patients With Solid Cancer: A Systematic Review and Meta-Analysis
Source: Front Oncol. 2021 Sep 23;11:691002. doi: 10.3389/fonc.2021.691002 (PMC8496897; doi:10.3389/fonc.2021.691002)
Supplement: Supplementary file 1 [file DataSheet_1.pdf]

supplementary Table 1 Results of quality assessment based on the Newcastle-ottawa scale for cohort studies

Study	Selection				Comparability		Outcome			Scores
	1	2	3	4	5A	5B	6	7	8	
Wang 2020	★	★	★		★		★	★		6
Sorich 2019	★	★	★		★		★	★	★	7
Mielgo 2019	★	★	★		★		★	★		6
Meyers 2019	★	★	★		★		★	★	★	7
Mazzaschi 2020	★	★	★		★		★	★	★	7
Kazandjian 2019	★	★	★				★	★	★	6
Herrera 2019	★	★	★		★		★	★		6
Ferreira 2019	★	★	★		★		★	★		6
Chen 2020	★	★	★		★		★	★		6
AI Darazi 2019	★	★	★		★		★	★	★	7
Santa 2020	★	★	★		★		★	★		6
Ruiz-Bañobre 2019	★	★	★		★		★			5

1 = representativeness of the exposed cohort; 2 = selection of the non-exposed cohort; 3 = ascertainment of exposure; 4 = demonstration that outcome of interest was not present at start of study; 5A = study controls for age, sex, marital status; 5B = study controls for any additional factors; 6 = assessment of outcome; 7 = follow-up long enough for outcomes to occur; 8 = adequacy of follow-up of cohorts. ★Asterisk means that the study is satisfied the item, no asterisk means the opposite situation.

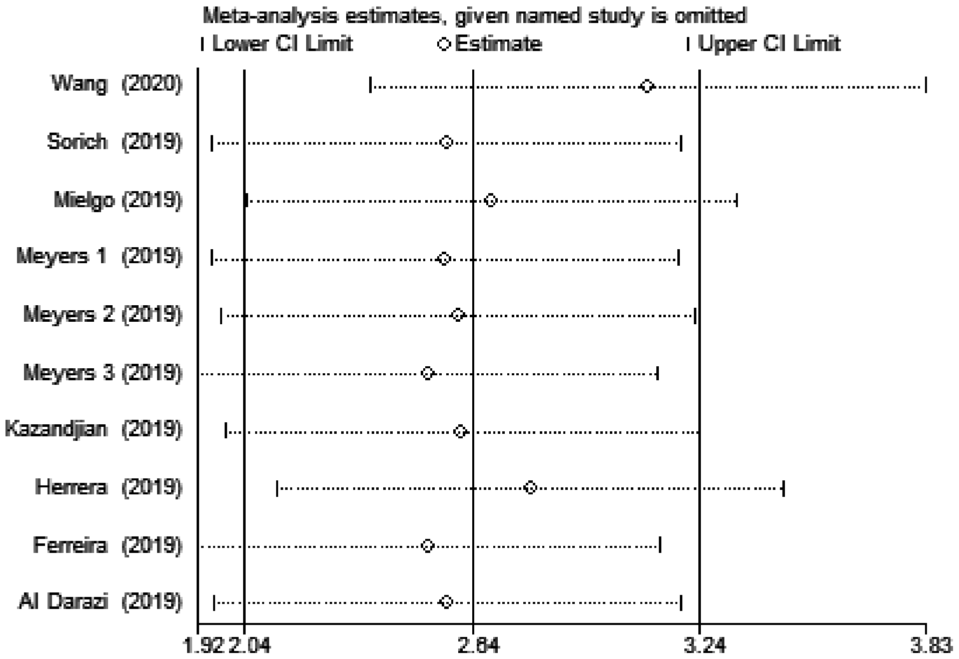

Supplementary Fig 1: sensitivity analysis of hazard ratio for overall survival in cancer patients after ICIs treatment (poor LIPI score groups vs good LIPI score groups)

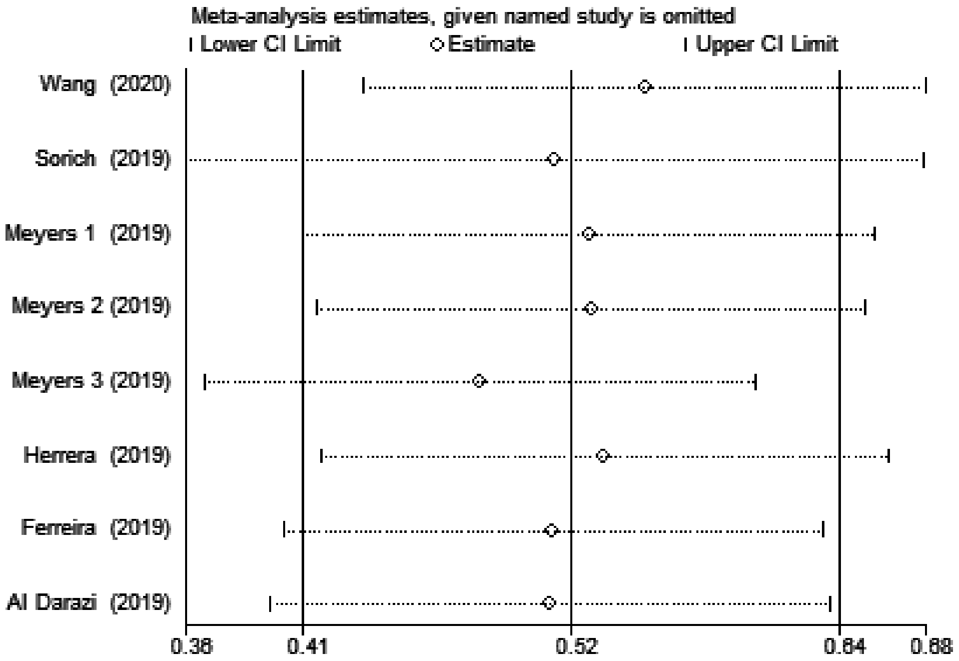

Supplementary Fig 2: sensitivity analysis of hazard ratio for overall survival in cancer patients after ICIs treatment (intermediate LIPI score groups vs good LIPI score groups)

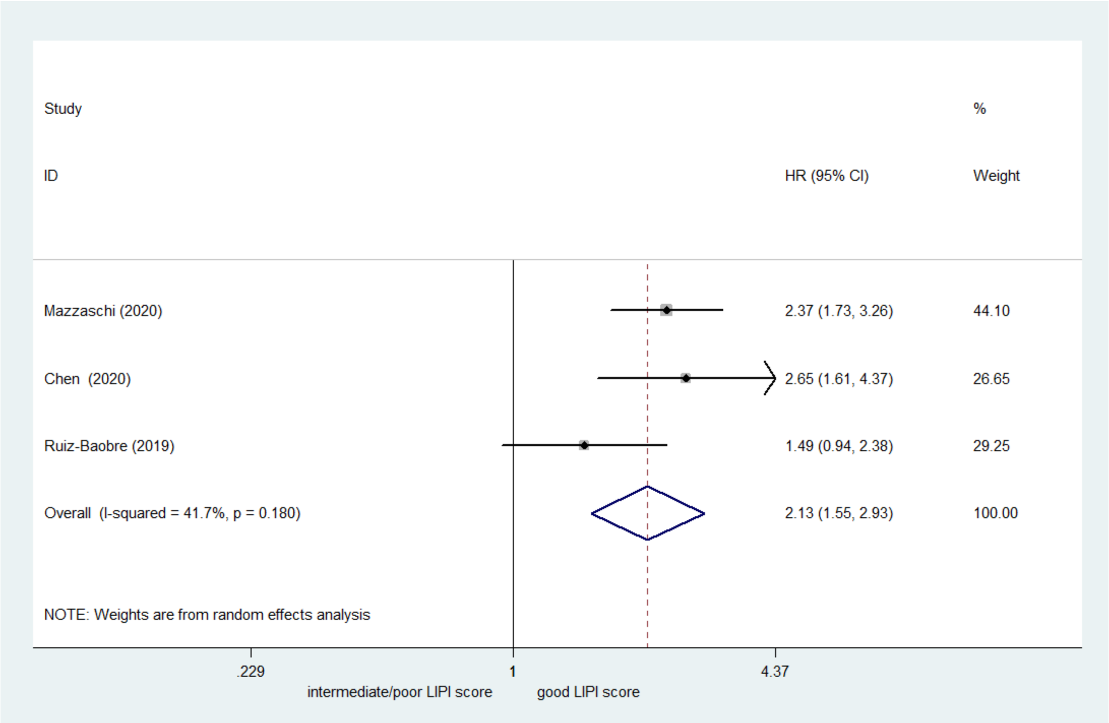

Supplementary Fig 3: Meta-analysis of PFS between intermediate/poor LIPI score group and good LIPI score group after receiving ICIs treatment (intermediate + poor LIPI score groups vs good LIPI score groups)

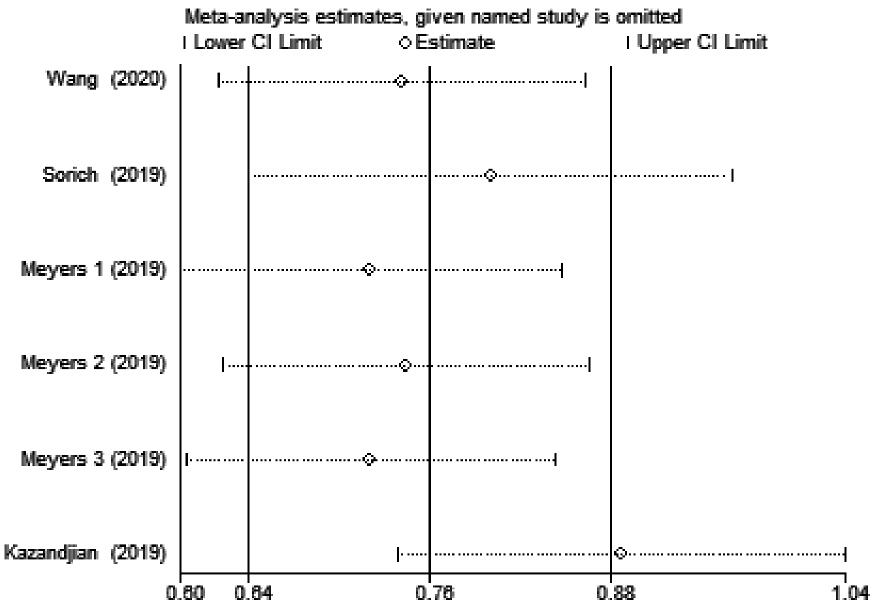

Supplementary Fig 4: sensitivity analysis of hazard ratio for PFS in cancer patients after ICIs treatment (poor LIPI score groups vs good LIPI score groups)

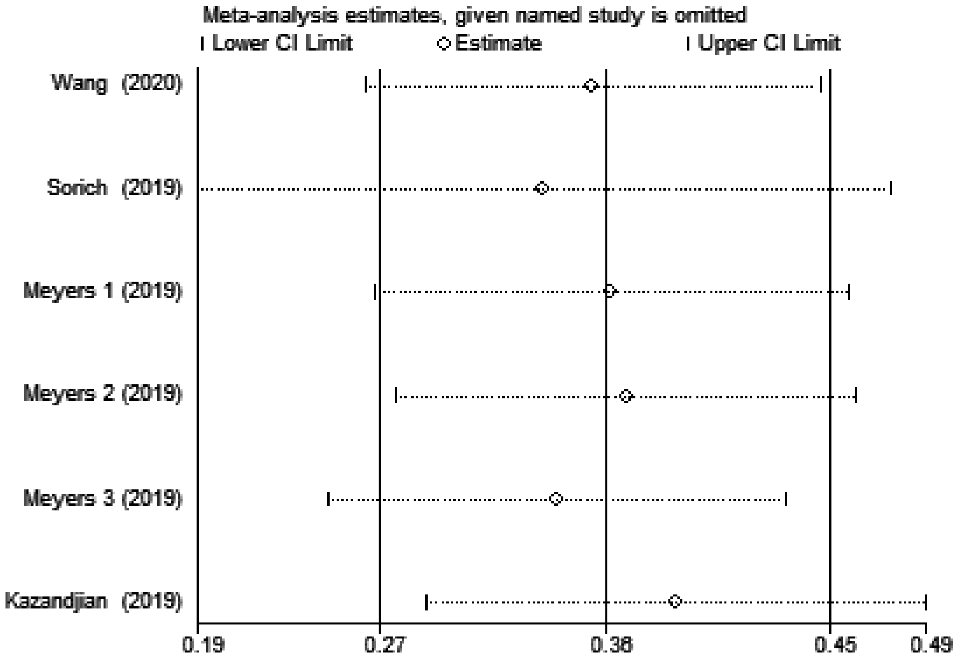

Supplementary Fig 5: sensitivity analysis of hazard ratio for PFS in cancer patients after ICIs treatment (intermediate LIPI score groups vs good LIPI score groups)

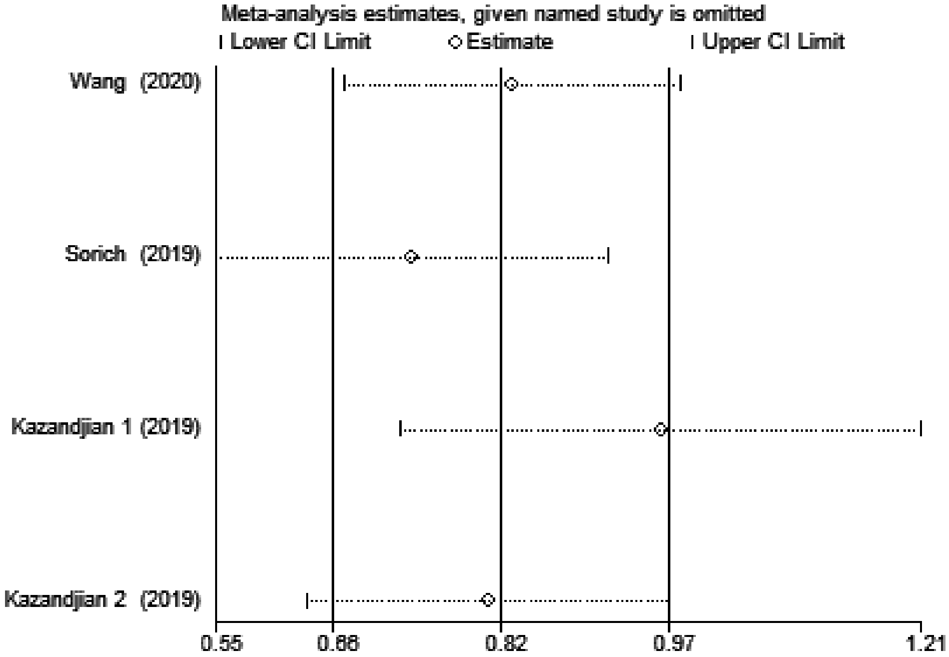

Supplementary Fig 6: sensitivity analysis of hazard ratio for OS in cancer patients after chemotherapy (poor LIPI score groups vs good LIPI score groups)

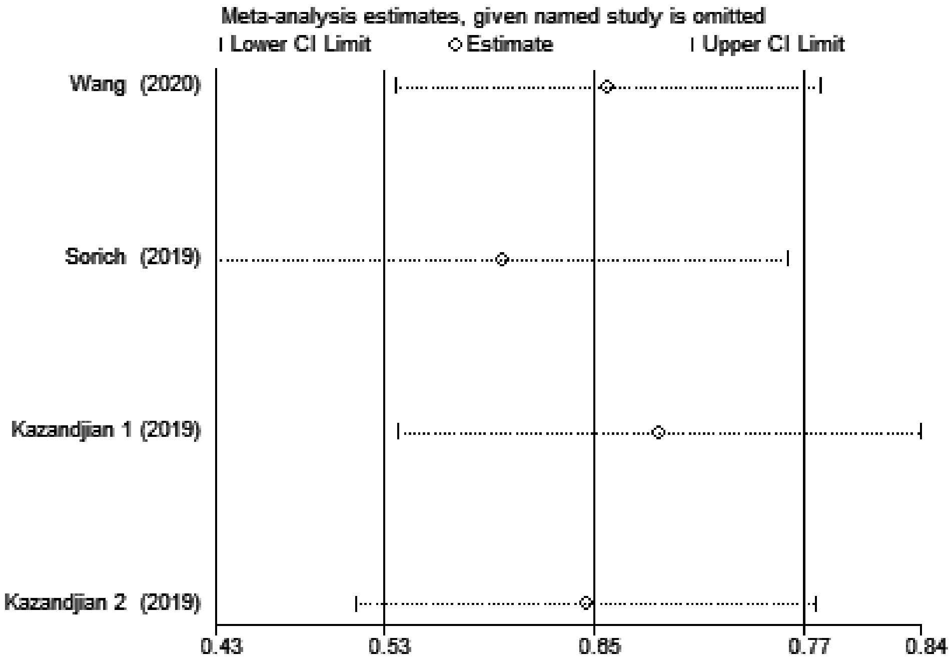

Supplementary Fig 7: sensitivity analysis of hazard ratio for PFS in cancer patients after chemotherapy (poor LIPI score groups vs good LIPI score groups)

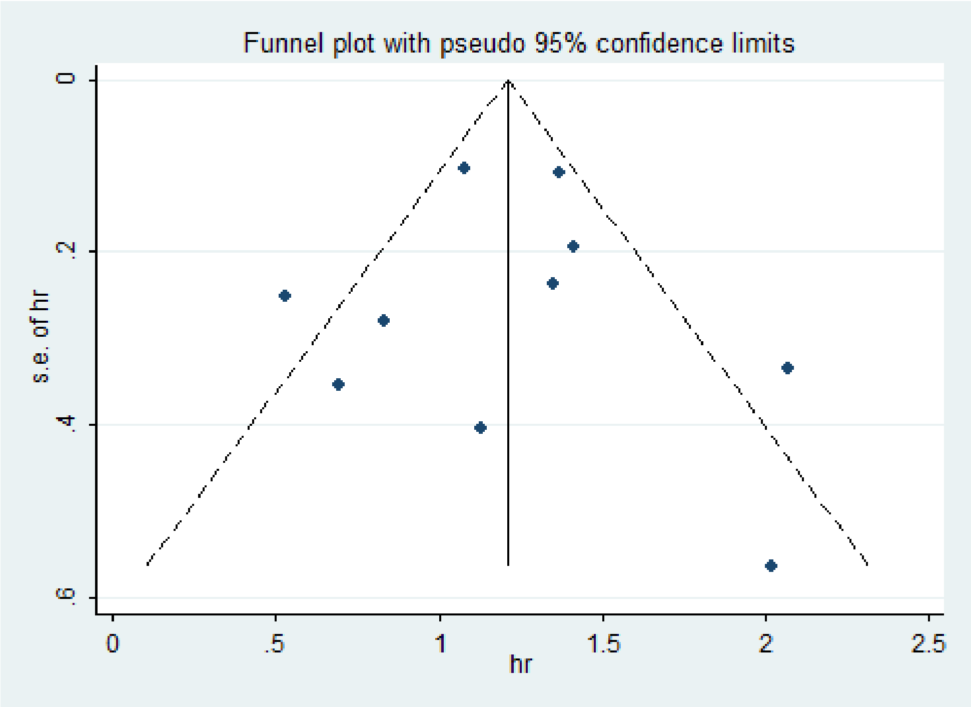

Supplementary Fig 8: Funnel plots of hazard ratio for OS in cancer patients after ICIs treatment

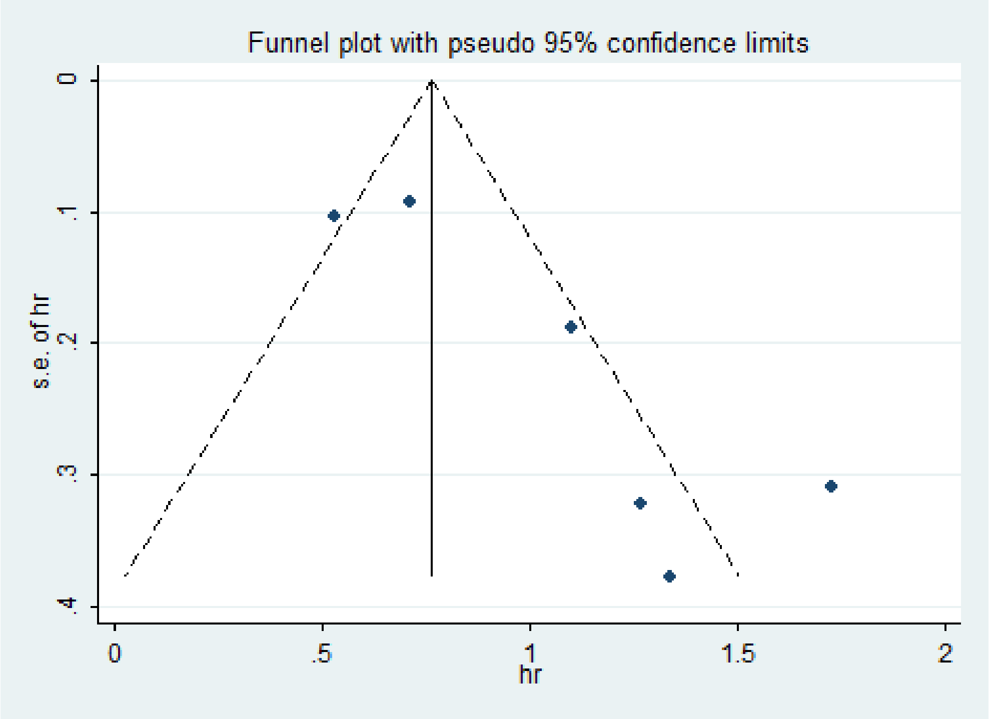

Supplementary Fig 9: Funnel plots of hazard ratio for PFS in cancer patients after ICIs treatment

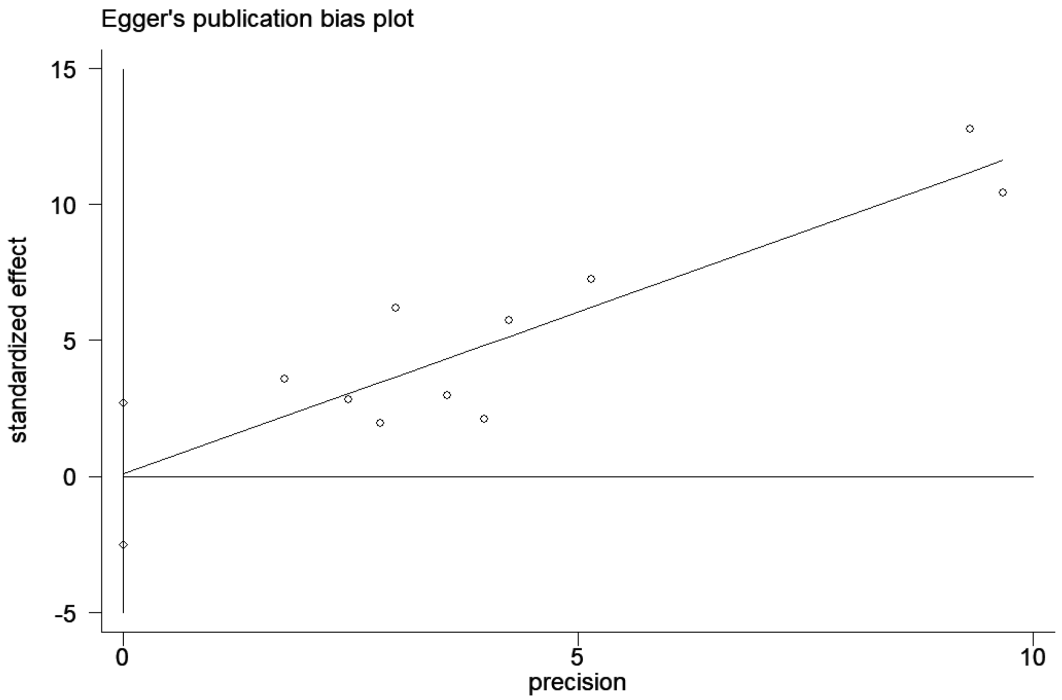

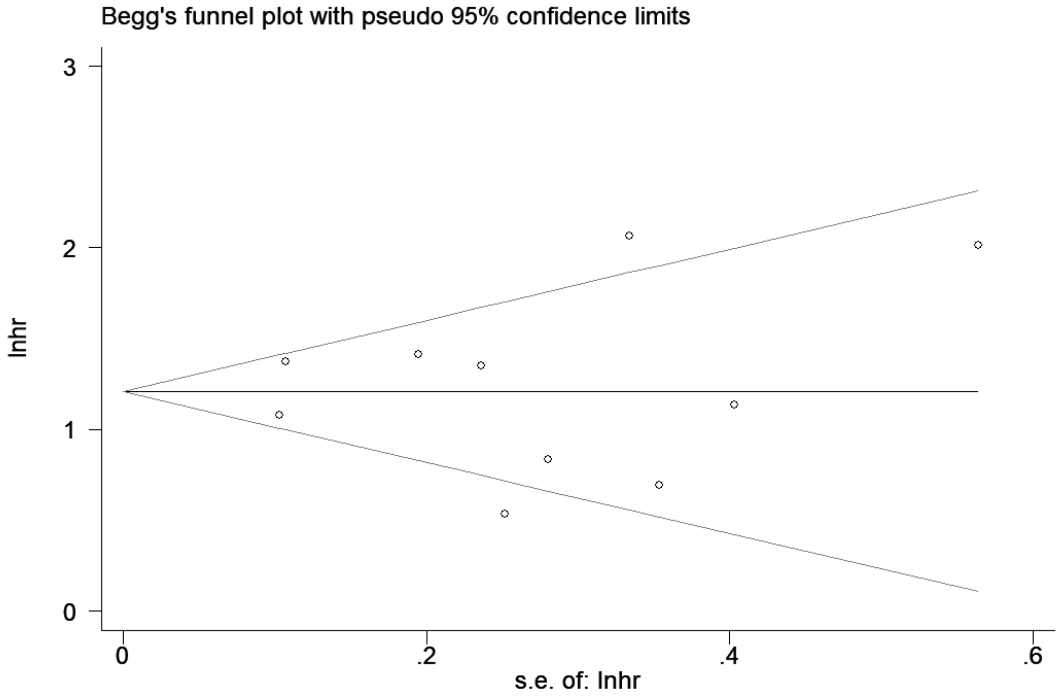

Supplementary Fig 10: Begg's and Egger's test of hazard ratio for OS in cancer patients after ICIs treatment (poor LIPI score groups vs good LIPI score groups)

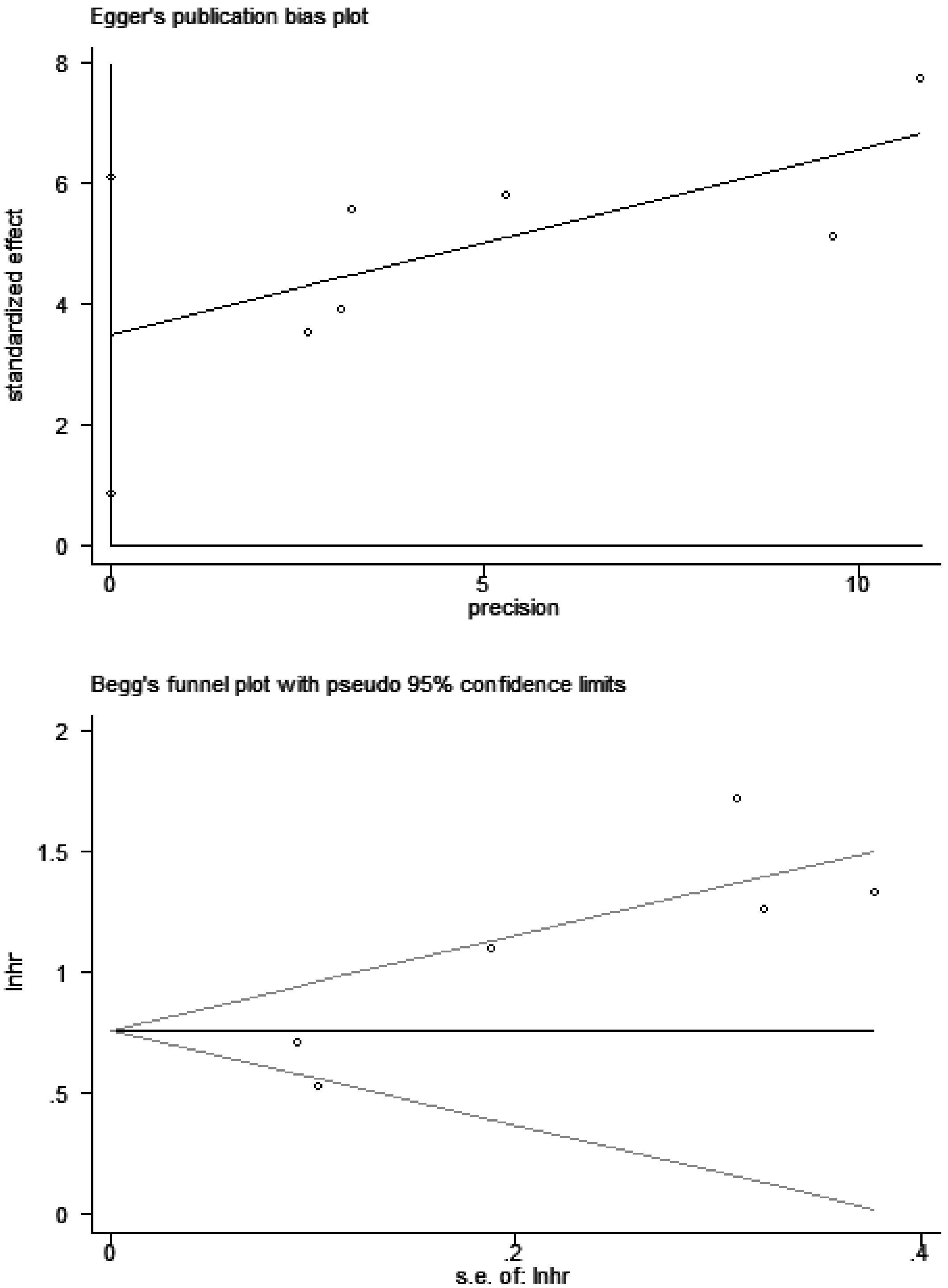

Supplementary Fig 11: Begg's and Egger's test of hazard ratio for PFS in cancer patients after ICIs treatment (poor LIPI score groups vs good LIPI score groups)

Supplementary Retrieval Methods

Search number	Query	Results
11	(((((((((((((PD-1[Title/Abstract]) OR (PD-L1[Title/Abstract])) OR (CTLA-4[Title/Abstract])) OR (nivolumab[Title/Abstract])) OR (atezolizumab[Title/Abstract])) OR (avelumab[Title/Abstract])) OR (durvalumab[Title/Abstract])) OR (ipilimumab[Title/Abstract])) OR (tremelimumab[Title/Abstract])) OR (pembrolizumab[Title/Abstract]))) OR (ICI*[Title/Abstract])) OR (immune checkpoint inhibitor*[Title/Abstract])) AND (("Neoplasms"[Mesh]) OR (((((((((((((((neoplasia[Title/Abstract]) OR (neoplasias[Title/Abstract])) OR (Neoplasm[Title/Abstract])) OR (Tumors[Title/Abstract])) OR (Tumor[Title/Abstract])) OR (Cancer*[Title/Abstract])) OR (Malignancy[Title/Abstract])) OR (Malignancies[Title/Abstract])) OR (Malignant Neoplasms[Title/Abstract])) OR (Malignant Neoplasm[Title/Abstract])) OR (Neoplasm, Malignant[Title/Abstract])) OR (Neoplasms, Malignant[Title/Abstract])) OR (Neoplasms, Benign[Title/Abstract])) OR (Benign Neoplasm[Title/Abstract])) OR (Benign Neoplasms[Title/Abstract])) OR (Neoplasm, Benign[Title/Abstract])))) AND ((lung immune prognostic index[Title/Abstract]) OR (LIPI[Title/Abstract]))	13
10	((((((((((((PD-1[Title/Abstract]) OR (PD-L1[Title/Abstract])) OR (CTLA-4[Title/Abstract])) OR (nivolumab[Title/Abstract])) OR (atezolizumab[Title/Abstract])) OR (avelumab[Title/Abstract])) OR (durvalumab[Title/Abstract])) OR (ipilimumab[Title/Abstract])) OR (tremelimumab[Title/Abstract])) OR (pembrolizumab[Title/Abstract]))) OR (ICI*[Title/Abstract])) OR (immune checkpoint inhibitor*[Title/Abstract])	42,766
7	("Neoplasms"[Mesh]) OR (((((((((((((((neoplasia[Title/Abstract]) OR (neoplasias[Title/Abstract])) OR (Neoplasm[Title/Abstract])) OR (Tumors[Title/Abstract])) OR (Tumor[Title/Abstract])) OR (Cancer*[Title/Abstract])) OR (Malignancy[Title/Abstract])) OR (Malignancies[Title/Abstract])) OR (Malignant Neoplasms[Title/Abstract])) OR (Malignant Neoplasm[Title/Abstract])) OR (Neoplasm, Malignant[Title/Abstract])) OR (Neoplasms, Malignant[Title/Abstract])) OR (Neoplasms, Benign[Title/Abstract])) OR (Benign Neoplasm[Title/Abstract])) OR (Benign Neoplasms[Title/Abstract])) OR (Neoplasm, Benign[Title/Abstract]))	4,242,314
6	(lung immune prognostic index[Title/Abstract]) OR (LIPI[Title/Abstract])	113

5	(((((((((((((((neoplasia[Title/Abstract]) OR (neoplasias[Title/Abstract])) OR (Neoplasm[Title/Abstract])) OR (Tumors[Title/Abstract])) OR (Tumor[Title/Abstract])) OR (Cancer*[Title/Abstract])) OR (Malignancy[Title/Abstract])) OR (Malignancies[Title/Abstract])) OR (Malignant Neoplasms[Title/Abstract])) OR (Malignant Neoplasm[Title/Abstract])) OR (Neoplasm, Malignant[Title/Abstract])) OR (Neoplasms, Malignant[Title/Abstract])) OR (Neoplasms, Benign[Title/Abstract])) OR (Benign Neoplasm[Title/Abstract])) OR (Benign Neoplasms[Title/Abstract])) OR (Neoplasm, Benign[Title/Abstract])	2,866,444
1	"Neoplasms"[Mesh]	3,382,544
